# Evaluation of preventable adverse drug reactions by implementation of the nationwide network of prospective drug utilization review program in Korea

**DOI:** 10.1371/journal.pone.0195434

**Published:** 2018-04-11

**Authors:** Jimin Lee, Yoojin Noh, Sukhyang Lee

**Affiliations:** College of Pharmacy, Ajou University, Suwon, South Korea; Public Library of Science, UNITED KINGDOM

## Abstract

**Background:**

A prospective Drug Utilization Review (DUR) program has been implemented in Korea to improve the quality and safety of medication use.

**Objective:**

To evaluate the influence of the DUR program in reducing incidence of preventable adverse drug reactions (pADRs).

**Methods:**

This study was performed using administrative data from the Health Insurance Review and Assessment Service (HIRA). The claims data for all adult patients with adverse drug events (ADE)-related diagnoses from 2009 to 2014 were obtained. Incidence rates of first-time and repeat pADRs prior to and after DUR program implementation were evaluated. Quarterly trends in incidence rates of overall ADE, allergic reactions, and ADRs were analyzed.

**Results:**

Data extraction covering the period from 2009 to 2014 led to the identification of 3,927,662 records. First-time pADR rates decreased gradually after implementation of the DUR program (change in slope: -0.016, p = 0.02). The program had a similar influence on repeat pADR rates (change in slope: -0.006, p≤0.01). The program did not decrease rates of first-time or repeat allergic reactions (change in slope: 0.018, p = 0.07 and 0.003, p = 0.04, respectively). In the cohort aged ≤65 years, first-time pADR rate reduction was significant (28.2% [27.1–29.3] in ≤18 years, and 19.8% [18.1–21.5] in 19–64 years). In contrast, first-time pADR rate was increased by 0.6% [-0.7–1.9] in patients ≥65 years.

**Conclusion:**

Implementation of the prospective DUR program effectively reduced the number of pADRs. In the future, to reduce non-preventable ADRs such as allergic reactions, provision of clinical information including allergy history should be added to the DUR program.

## Introduction

An adverse drug event (ADE) is defined as “an injury resulting from medical intervention related to a drug” [1]. The term encompasses harms that occur in any health care setting that are directly due to the drug including but not limited to medication errors, adverse drug reactions (ADRs), allergic reactions, and overdoses [2].

The clinical and economic burdens of ADEs are significant. Thus, it is critical to minimize the potential consequences of ADEs. Although a large majority of medical errors and ADRs are preventable, numerous studies have reported that ADRs are associated with clinically significant morbidity and mortality, and are a serious threat to public health [3–5]. A review of 25 studies including 106,586 hospitalized patients found that up to 15.7% of hospitalized patients experienced ADRs [3]. A study focused on more vulnerable populations reported that the overall incidence of ADRs was 9.5% and 1.5% in hospitalized and outpatient children, respectively [4]. Another study reviewed ADEs in the elderly, and showed that 23.1% of emergency department admissions were found to be ‘probably’ or ‘possibly’ medication-related (13.1% and 10.0%, respectively) [5]. Therefore, medication-related adverse events leading people to seek medical care are common in all populations, including special populations.

To reduce potential ADEs and their consequences, many types of quality assurance programs have been developed and introduced [6–8]. In South Korea, a prospective Drug Utilization Review (DUR) program, a systematic review program integrated with electronic medical records (EMRs) and electronic insurance claims submission systems, has been in use since December 2010 to improve the quality and safety of medication use [9, 10]. It is reported that 99.4% of South Korean outpatient health care facilities are currently utilizing the program to alert preventable ADRs (pADRs) [10]. However, despite the program’s purpose, no study has evaluated its influence in reducing the incidence of pADRs since its implementation.

We utilized the national health insurance claims database to evaluate the influence of the DUR program in reducing pADR incidence by quantitatively studying trends in ADE episodes leading people to seek medical care.

## Material and methods

### The DUR program in Korea

The DUR program in Korea uses a concurrent process to improve the quality of medication use and health outcomes with information technology that creates a shared network between the healthcare providers and the computer server of the Health Insurance Review and Assessment Service (HIRA) since December 2010. It involves monitoring and reviewing drug use to evaluate appropriateness based on best medication use criteria. The program implemented interventions to improve medication use and overall patient care. It monitors 33,550 drug products and 2,070 ingredients for duplicate prescription, drug-drug interaction, contraindication due to pregnancy and/or age, potential inappropriate medication for the elderly, and dose and duration of drug regimen [10]. The criteria and list of drugs in the DUR program are updated annually. As an example, the DUR program reviewed 1,121,234,782 prescriptions that fell within DUR modules in the year 2014 [10]. The DUR program sends alert messages to healthcare providers for prevention of ADR for patient safety.

### Identification of relevant ICD-10 codes

ADE-related diagnosis codes were adapted from a study conducted by Stausberg and colleagues [11]. In this study, a total of 505 International Classification of Diseases (ICD-10) codes were grouped into seven categories: A.1 “induced by medication”; A.2 “induced by medication or other causes”; B.1 “poisoning by medication”; B.2 “poisoning by or harmful use of medication or other causes”; C “ADE very likely”; D “ADE likely”; and E “ADE possible”. Of these, we selectively limited our analysis utilizing ICD-10 codes in categories A1, A2, B1, B2, and C so that identified ADEs are definite. Additionally, we distinguished a classification for allergic responses, since the DUR program does not include a module that alerts known drug allergy history.

### Data source

This was a population-based retrospective cohort study using administrative data from the HIRA claims database. The database consists of information on the entire health care system used by approximately 50 million Koreans, and covered by National Health Insurance (NHI). The NHI program is a universal health care system that allows beneficiaries to access any medical facilities in Korea with low or no co-payment. Those who are unable to afford co-payments are covered by the national insurance and exempted from co-payment. Therefore, the HIRA database contains records of all Koreans regardless of socioeconomic status.

The database contains clinic and hospital visit records that record patient information including age, sex, diagnosis, medical procedure and services, type of health care institution, date of visit, admission and discharge, length of hospitalization, and medical specialty. The database also contains pharmacy records regarding prescribed medications, such as brand and generic names, strength, quantity, route of administration, single administration doses, total daily doses, and prescription dates. The diagnoses are coded using the sixth revision of the Korean Standard Classification of Diseases, which reflects the ICD-10 codes. Prior to providing the data, HIRA encrypted the original identification of each patient to protect privacy. The study was evaluated and approved by the institutional review board of Ajou University (IRB No. 201507-HR-BM-001-01). Patient informed consent was exempted since all patient information was fully encrypted.

### Patient population

**Fig 1:**
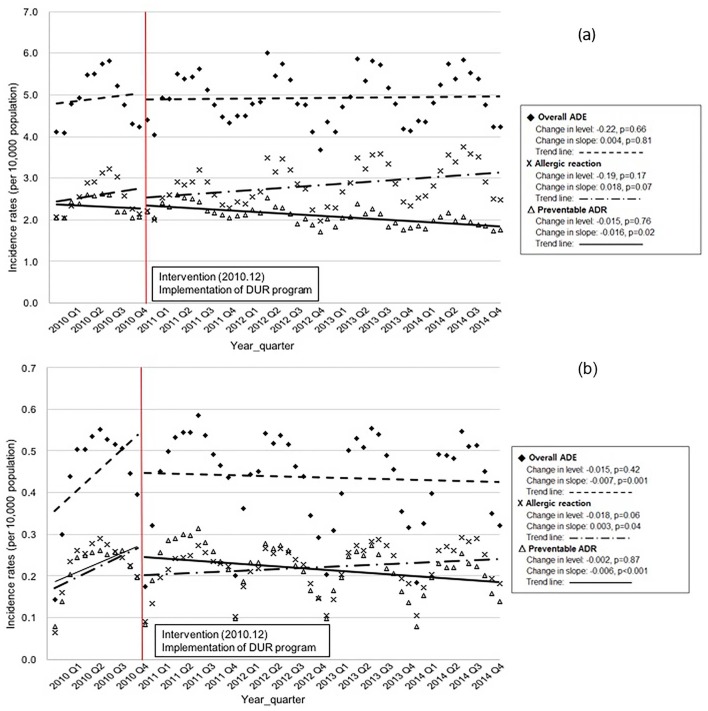
(a) Trends in incidence rates of first-time ADE, allergic reactions, and preventable ADR. (b) Trends in incidence rates of repeat ADE, allergic reactions, and preventable ADR.

Claims data for all adult patients (≥18 years of age) with ICD-10 codes in categories A1, A2, B1, B2 and C from January 1, 2009 to December 31, 2014 were obtained. A first-time event was defined as an event occurring for the first time during the study period with no prior events for a minimum of one year. We excluded events occurring in 2009 to identify the first one that occurred in 2010. If the same type of event recurred within 30 days, the events were considered as a single event. On the other hand, if an ADE or an ADR occurred at least 30 days after the first event, it was considered as a repeat event. For overall trends in incidence rates, all events from 2010 to 2014 were included (Fig 1). For analysis of the influence of DUR program implementation with regards to sex, age, types of health care system, and most commonly observed diagnoses, the data was refined to ADRs occurring in 2010 and in 2014. To evaluate incidence rates and relative reduction, we excluded data from 2011 to 2013 (the so-called “adaptation period’) since the program was implemented gradually during this period.

### Statistical analysis

Patient characteristics before and after DUR program implementation are compared using descriptive statistics. Events related to allergic reactions and pADRs collectively comprise the ADE dataset. Quarterly trends in incidence rates of first-time and repeat ADEs, allergic reactions, and ADRs are analyzed. A two-phase interrupted time series design with segmented linear regression analysis is used to evaluate the longitudinal effects of system implementation: pre-DUR program implementation (phase I; January 2010–December 2010), post-DUR program implementation (phase II; January 2011–December 2014). Seasonal variations are statistically normalized and autocorrelation is corrected to avoid underestimated standard errors and overestimated significance of the effects of an intervention [12]. Frequencies of first-time and repeat events that occurred in 2010 (pre-DUR) and 2014 (post-DUR) are compared in the form of incidence rates per 10,000 population (number of incidents divided by number of persons covered by NHI each year). Subgroup analyses are performed according to sex, age, types of health care system, and most commonly observed diagnoses. All analyses are carried out with SAS statistical software (version 9.4 for Windows: SAS Institute, Inc., Cary, NC, USA), with all p values less than 0.05 considered to be statistically significant.

## Results

Data extraction from the HIRA database covering the years 2009 through 2014 identified 3,927,662 records with a primary diagnosis of an ADE. The incidence rates of first-time ADEs were 58.9, 58.8, 58.5, 59.1 and 59.8 per 10,000 population in 2010, 2011, 2012, 2013, and 2014, respectively. On the other hand, 5.4, 5.6, 5.1, 5.2 and 5.1 repeat ADEs occurred in every 10,000 population.

Demographic data and clinical characteristics are presented in Table 1. pADRs represented 634,324 of the first-time episodes (43.3%) that occurred during the 5-year period (2010–2014). Of these, 43.1% were male patients. The mean age of these patients was 47.0 years (standard deviation (SD) = 20.2) and the cohort aged ≥65 years represented 19.8% of the study population. A total of 525,412 pADRs (51.6%) events caused primary care provider visits, while 96,413 events (15.2%) led to treatment at tertiary hospitals.

**Table 1:** Characteristics of study subjects who experienced adverse drug events in 2010–2014.

	First ADEs		Repeat ADEs	
	Preventable ADRs	Allergic reactions	Preventable ADRs	Allergic reactions
	(n = 634,324)	(n = 831,080)	(n = 64,816)	(n = 65,588)
Sex				
Male, n (%)	273,411 (43.1)	357,584 (43.0)	28,486 (43.9)	27,800 (42.4)
Female, n (%)	360,913 (56.9)	473,496 (57.0)	36,330 (56.1)	37,788 (57.6)
Age, years, Mean ± SD	47.0 ± 20.2	40.7 ± 22.1	52.6 ± 18.6	43.4 ± 23.2
≤18, n (%)	60,079 (9.5)	163,962 (19.7)	2,942 (4.5)	12,791 (19.5)
19–64, n (%)	448,955 (70.8)	548,235 (66.0)	44,332 (68.4)	39,819 (60.7)
≥65, n (%)	125,290 (19.8)	118,883 (14.3)	17,542 (27.1)	12,978 (19.8)
Types of health care system				
Tertiary hospitals, n (%)	96,413 (15.2)	101,659 (12.2)	15,958 (24.6)	12,203 (18.6)
Secondary hospitals, n (%)	210,864 (33.2)	195,168 (23.5)	20,958 (32.3)	10,329 (15.7)
Primary care providers, n (%)	327,047 (51.6)	534,253 (64.3)	27,900 (43.0)	43,056 (65.6)
Admission type				
Inpatients, n (%)	48,508 (7.6)	12,471 (1.5)	2,866 (4.4)	717 (1.1)
Emergency room, n (%)	60,404 (9.5)	14,289 (1.7)	1,477 (2.3)	490 (0.7)
Outpatients, n (%)	525,412 (82.8)	804,320 (96.8)	60,473 (93.3)	64,381 (98.2)
CCI, Mean ± SD	0.05 ± 0.27	0.03 ± 0.18	0.10 ± 0.38	0.07 ± 0.30
Length of hospital stay, Mean ± SD	3.1 ± 5.7	1.4 ± 2.0	2.4 ± 5.3	1.4 ± 2.2

Abbreviations: ADE, adverse drug events; ADR, adverse drug reaction; CCI, Charlson Comorbidity Index; SD, standard deviation

The repeat/first-time pADR event ratio was 9.8. Among the people who experienced first-time pADRs, the same types of ADR occurred in 64,816 cases (43.9% male; mean [SD] age, 52.6 [18.6] years). Among these people, 27,900 (43.0%) sought medical care from primary care physicians while 15,958 (24.6%) were treated at tertiary hospitals.

Fig 1 shows the quarterly incidence rates of total ADEs, allergic reactions, and pADRs with seasonal variation. First-time pADR rates did not decrease immediately. Rather, they decreased gradually after implementation of the DUR program (change in slope: -0.016, p = 0.02). The DUR program had a similar influence on repeat pADR rates (change in slope: -0.006, p≤0.01). On the other hand, the program did not decrease rates of first-time or repeat allergic reactions (change in slope: 0.018, p = 0.07 and 0.003, p = 0.04, respectively).

Table 2 provides a comparison of pADR rates during pre- and post-DUR program implementation according to sex, age group, and type of health care system. The relative reduction of first-time pADRs in both male and female populations (16.7% [95% CI: 15.1–18.2] and 17.2% [15.6–18.9] were significant. In the population aged ≤65 years, first-time pADR rate reductions were significant (28.2% [27.1–29.3] in ≤18 years and 19.8% [18.1–21.5] in 19–64 years). By contrast, first-time pADR rates increased by 0.6% [-0.7–1.9] in the population ≥65 years. The relative reductions of repeat pADR rates were 23.1% [22.5–23.6], 20.8% [19.8–21.8], and 1.6% [0.8–2.4] in ≤18, 19–64, and ≥65 years, respectively. Among the different types of health care system, primary care providers were the most significantly influenced by DUR program implementation, with first-time and repeat pADRs reduced by 28.9% [27.3–30.5] and 34.8% [33.9–35.7], respectively.

**Table 2:** Incidence and relative reduction of preventable ADR by baseline characteristics.

		First-time pADRs			Repeat pADRs		
		No. of incidence		Relative reduction^b^	No. of incidence		Relative reduction^b^
		(incidence rates^a^)		(95% CI)	(incidence rates^a^)		(95% CI)
		Pre-DUR	Post-DUR		Pre-DUR	Post-DUR	
		(year 2010)	(year 2014)		(year 2010)	(year 2014)	
Overall		135,863 (27.8)	116,041 (24.1)	-17.0 (-18.8;-15.2)	13,424 (2.7)	11,572 (2.3)	-16.2 (-17.3;-15.1)
Sex	Male	58,591 (12.0)	50,237 (10.0)	-16.7 (-18.2;-15.1)	5,928 (1.2)	4,992 (1.0)	-18.1 (-19.0;-17.3)
	Female	77,272 (15.8)	65,804 (13.1)	-17.2 (-18.9;-15.6)	7,496 (1.5)	6,580 (1.3)	-14.7 (-15.6;-13.7)
Age	≤18	13,628 (2.8)	10,067 (2.0)	-28.2 (-29.3;-27.1)	648 (0.1)	513 (0.1)	-23.1 (-23.6;-22.5)
	19–64	97,849 (20.0)	80,732 (16.0)	-19.8 (-21.5;-18.1)	9,471 (1.9)	7,714 (1.5)	-20.8 (-21.8;-19.8)
	≥65	24,386 (5.0)	25,242 (5.0)	0.6 (-0.7;1.9)	3,305 (0.7)	3,345 (0.7)	-1.6 (-2.4;-0.8)
Types of health care	Tertiary hospital	18,469 (3.8)	19,245 (3.8)	1.3 (0.1;2.5)	2,945 (0.6)	3,058 (0.6)	0.9 (0.1;1.7)
	Secondary hospital	41,918 (8.6)	41,577 (8.3)	-3.6 (-5.1;-2.1)	3,966 (0.8)	4,142 (0.8)	1.5 (0.7;2.3)
	Primary care provider	75,476 (15.4)	55,219 (11.0)	-28.9 (-30.5;-27.3)	6,513 (1.3)	4,372 (0.9)	-34.8 (-35.7;-33.9)

Abbreviations: pADR, preventable adverse drug reaction; CI, confidence interval; DUR, drug utilization review.

^**a**^Number of incidents divided by total population in 2010 or 2014. Units: events per 10,000 population

^**b**^Units: %

Table 3 reports the five most frequently observed ICD-10 codes related to pADRs and the relative reduction of each diagnosis code after DUR program implementation. In first-time pADRs, K71 [Drug-induced idiosyncratic liver disease] was the most frequent diagnosis (36.5%), followed by L279 [Dermatitis due to unspecified substances taken internally] (13.0%) and L278 [Dermatitis due to other substances taken internally] (7.6%). K71 accounted for 42.8% of repeat pADRs, followed by L279 (8.5%), and G253 [Drug-induced myoclonus] (5.2%). Both first-time and repeat pADR rates related to K71 were significantly reduced after implementation of the DUR program (relative reduction: 25.2% [23.7–26.7] and 19.6% [18.7–20.5], respectively. The highest relative reduction of pADR rates was related to L279: 31.9% [30.7–33.0] for first-time events and 65.7% [65.1–66.3] for repeat events.

**Table 3:** Incidence and relative reduction of preventable ADR for top 5 most frequently observed diagnoses.

	ICD-10	Description	No. of incidence		Relative reduction^b^
			(incidence rates^a^)		(95% CI)
			Pre-DUR	Post-DUR	
			(year 2010)	(year 2014)	
First-time pADRs	K71	Drug-induced idiosyncratic liver disease	51,349 (10.5)	39,508 (7.9)	-25.2 (-26.7;-23.7)
	L279	Dermatitis due to unspecified substances taken internally	19,463 (4.0)	13,644 (2.7)	-31.9 (-33.0;-30.7)
	L278	Dermatitis due to other substances taken internally	10,178 (2.1)	9,325 (1.9)	-10.9 (-12.0;-9.9)
	T42	Poisoning by antiepileptic, sedative-hypnotic and antiparkinsonism drugs	6,330 (1.3)	5,669 (1.1)	-13.0 (-13.9;-12.0)
	T50	Poisoning by diuretics and other and unspecified drugs, medicaments and biological substances	5,409 (1.1)	5,590 (1.1)	0.5 (-0.4;1.3)
Repeat pADRs	K71	Drug-induced idiosyncratic liver disease	5,796 (1.2)	4,794 (1.0)	-19.6 (-20.5;-18.7)
	L279	Dermatitis due to unspecified substances taken internally	1,716 (0.4)	606 (0.1)	-65.7 (-66.3;-65.1)
	G253	Drug-induced myoclonus	911 (0.2)	541 (0.1)	-42.3 (-42.8;-41.7)
	D70	Drug-induced neutropenia	432 (0.1)	817 (0.2)	83.8 (83.3;84.3)
	E242	Drug-induced Cushing’s syndrome	415 (0.1)	382 (0.1)	-10.5 (-11.0;-10.0)

Abbreviations: pADR, preventable adverse drug reaction; CI, confidence interval; DUR, drug utilization review; ICD, International Classification of Diseases.

^**a**^Number of incidents divided by total population in 2010 or 2014. Units: events per 10,000 population

^**b**^Units: %

## Discussion

The present study provides a comprehensive, population-based evaluation of the influence of DUR program implementation on the incidence of first-time and repeat pADRs. Our data demonstrate that 59 out of every 10,000 individuals experience adverse events due to medications at least once each year, and of those, 26 individuals experience preventable ones. The DUR program had a significant impact on healthcare as demonstrated by decreased rates of pADRs while rates of allergic reactions unchanged or trended up. Since the DUR program does not include a module that alerts drug allergy history, this was as expected. The HIRA agency is currently making an effort to establish a module related to allergy alerts which requires a complex algorithm. Instead, hospitals with a pharmacovigilance center currently have an institution-level DUR program to alert the ADR risk for patients with allergy history using an ADR database.

A few studies have evaluated the impact of the DUR program on clinical outcomes, although results have been heterogeneous [7, 13]. Hennessy and colleagues retrospectively studied the effect of DUR implementation on the rate of exceptions, which were defined as a violation of criteria, and clinical outcomes for patients with exceptions using Medicaid data [7]. They found no reduction in the rate of exceptions coincident with DUR implementation (rate increase, 0.064 exceptions per 1,000 prescriptions per month; 95%, -0.006–0.133). Implementation of the DUR program also had no effect on the incidence of hospitalization for all causes (odd ratio (OR), 0.99; 95% CI, 0.98–1.00). Yet, these results were not comprehensive, since the analysis was limited to three criteria (drug-drug or drug-disease interactions, and therapeutic duplication) and other criteria such as excessive dose, insufficient dose, and excessive medication duration were purposely omitted.

A case-controlled retrospective study evaluating the impact of DUR implementation on physician spillover effect on future prescription following pharmacist’s intervention reported that a physician in a case group was eight times less likely to receive repeated interventions than a physician in a control group (2.2% and 18.2%, p<0.001, respectively) [13]. The shortcoming of this study was that the results were restricted to its impact on subsequent physician prescribing patterns, rather than directly correlating their interventions with clinical outcomes.

There are also a few studies evaluating effects of the DUR program in Korea since implementation. However, previous studies have focused on its effectiveness in cost-saving or in reducing the number of inappropriately prescribed medications [9, 14–16] rather than its consequences such as hospitalization due to medication misuse.

In contrast, we evaluated the DUR program’s direct effects on reducing specific negative clinical outcomes; the incidence of preventable adverse events due to medications. Since the program is not designed to capture non-preventable ADEs such as allergic reactions, we used rates of total ADEs and of allergic reactions as controls. As shown in Fig 1A, first-time incidence rates of total ADEs and allergic reactions trended up (change in slopes: 0.004, p = 0.81 and 0.018, p = 0.07) despite implementation of the DUR program. The rate of repeat allergic reactions was not reduced (change in slope: 0.003, p = 0.04) (Fig 1B). The most likely reason that first-time and repeat allergic reaction rates were increased is that there have been increased awareness and improved recording of drug allergies and consumption of drugs while no action has been taken to reduce the risk of allergic reaction. Thus, a marked rise in the incidence rates of allergic reaction appears unrelated to the implementation of the DUR program. Our study results strongly support the government agency to include the allergy alert module in the DUR program.

Conversely, both first-time and repeat pADR rates were significantly decreased (change in slopes: -0.016, p = 0.02 and -0.006, p≤0.001, respectively) upon DUR program implementation. In addition, when comparing incidence rates during pre- and post-DUR periods (Tables 2 and 3), post-DUR rate reductions remain significant (17.0% [15.2–18.8] for first-time pADRs, and 16.2% [15.1–17.3] for repeat pADRs).

ADRs are recognized as a major cause of morbidity, especially in the elderly. It has been reported that 36.7% of total prescriptions to elderly patients visiting ambulatory care were inappropriately prescribed [14]. This was confirmed by a regional pharmacovigilance center of the Korean Pharmaceutical Association, as one-third of reported ADRs were experienced by elderly patients [17]. Unfortunately, we found that elderly patients were the least benefitted by the DUR program’s effect on pADR rates. In fact, the rate of first-time pADRs in the population ≥65 years remained unchanged. The most plausible reason that the program was not effective in reducing pADRs in this population is that the criterion specifically designed to prevent inappropriate medication use in the elderly was absent at the time of program implementation. Despite the well-known fact that the elderly are at higher risk of experiencing ADRs because of multiple morbidities and polypharmacy than the general population, the criterion “precautions for use in the elderly” was not introduced until October 2015. Thus, the observed pADR rate reductions did not reflect the addition of this new criterion. Currently, the criterion has nineteen medications on its list, and most of them are known to be sedating and to increase risk of cognitive impairment (S1 Table). While it is clear that these medications should be avoided in the elderly, the list also needs to be expanded, as many researchers have suggested a wide range of potentially inappropriate medication uses in this population [18–20].

Liver damage was one of the disease manifestations most frequently identified as medication-induced in many studies [21, 22]. In the present analysis, the most commonly observed diagnoses were associated with liver damage, consistent with previous findings. We found that implementation of the DUR program effectively reduced the incidence of both first-time and repeat pADRs related to liver symptoms (relative reduction: 25.2% [23.7–26.7] and 19.6% [18.7–20.5], respectively).

To our knowledge, this is the first study to evaluate the effectiveness of implementation of the nationwide, prospective DUR program in reducing first-time and repeat pADRs. While recognizing its financial benefits by reducing total health care system and medication usage is important, our analyses demonstrated that the DUR program is able to detect pADRs before medication is dispensed and prevent potentially fatal medical consequences.

However, our study had several limitations. First, although pharmacists are encouraged to enter the over-the-counter medications into the DUR program for comprehensive medication review, it is not mandatory. Thus, it is possible that the DUR is limited to prescription medications. Second, when pharmacists’ are dispensing medications, they are unable to review the appropriateness of prescribed medications since the program does not provide clinical information. Therefore, the effect of the DUR program may have been underestimated in preventing adverse medical results.

In conclusion, implementation of the prospective DUR program was effective in reducing the incidence of pADRs. Continuous updates of criteria in the DUR program is encouraged to minimize pADRs. In addition, in order to reduce non-preventable adverse drug reactions, such as allergic reactions, providing clinical information including allergy history should be established in the DUR program.

## Supporting information

S1 Table List of medications under the criterion “precaution for use in the elderly”. (DOCX)

## References

[1] Kohn LT, Corrigan J, Donaldson MS. To err is human: building a safer health system. Washington, D.C.: National Academy Press; 2000 xxi, 287 p. p. 25077248

[2] Ducoffe AR, Baehr A, Pena JC, Rider BB, Yang S, Hu DJ. Adverse Drug Event Prevention: 2014 Action Plan Conference. American journal of medical quality: the official journal of the American College of Medical Quality. 2016;31(5):476–85. doi: 10.1177/1062860615588105 . 26024666 10.1177/1062860615588105

[3] Kongkaew C, Noyce PR, Ashcroft DM. Hospital admissions associated with adverse drug reactions: a systematic review of prospective observational studies. The Annals of pharmacotherapy. 2008;42(7):1017–25. doi: 10.1345/aph.1L037 . 18594048 10.1345/aph.1L037

[4] Impicciatore P, Choonara I, Clarkson A, Provasi D, Pandolfini C, Bonati M. Incidence of adverse drug reactions in paediatric in/out-patients: a systematic review and meta-analysis of prospective studies. British journal of clinical pharmacology. 2001;52(1):77–83. doi: 10.1046/j.0306-5251.2001.01407.x . 11453893 10.1046/j.0306-5251.2001.01407.x PMC2014499

[5] Laatikainen O, Sneck S, Bloigu R, Lahtinen M, Lauri T, Turpeinen M. Hospitalizations Due to Adverse Drug Events in the Elderly-A Retrospective Register Study. Frontiers in pharmacology. 2016;7:358 doi: 10.3389/fphar.2016.00358 . 27761112 10.3389/fphar.2016.00358 PMC5051318

[6] Armstrong EP, Proteau D. Retrospective drug utilization review software systems: perspectives of state Medicaid DUR directors. The Annals of pharmacotherapy. 1996;30(10):1088–91. doi: 10.1177/106002809603001004 . 8893113 10.1177/106002809603001004

[7] Hennessy S, Bilker WB, Zhou L, Weber AL, Brensinger C, Wang Y, et al Retrospective drug utilization review, prescribing errors, and clinical outcomes. Jama. 2003;290(11):1494–9. doi: 10.1001/jama.290.11.1494 . 13129990 10.1001/jama.290.11.1494

[8] Fulda TR, Collins T, Kuhle J, Devereaux DS, Zuckerman IH. Medicaid drug utilization review annual reports for federal fiscal year 1999: looking back to move forward. Journal of the American Pharmacists Association: JAPhA. 2004;44(1):69–74. . 14965156 10.1331/154434504322713255

[9] Yang JH, Kim M, Park YT, Lee EK, Jung CY, Kim S. The effect of the introduction of a nationwide DUR system where local DUR systems are operating—The Korean experience. International journal of medical informatics. 2015;84(11):912–9. doi: 10.1016/j.ijmedinf.2015.08.007 . 26363001 10.1016/j.ijmedinf.2015.08.007

[10] Lee S, Hong S, Kim J, Ye Y, Lee JY, Ji E, et al. Assessment and development plan of drug utilization review program. Health Insurance Review and Assessment Service., 2016 Contract No.: G000DE1-2016-35.

[11] Stausberg J, Hasford J. Identification of adverse drug events: the use of ICD-10 coded diagnoses in routine hospital data. Deutsches Arzteblatt international. 2010;107(3):23–9. doi: 10.3238/arztebl.2010.0023 . 20140170 10.3238/arztebl.2010.0023 PMC2816787

[12] Wagner AK, Soumerai SB, Zhang F, Ross-Degnan D. Segmented regression analysis of interrupted time series studies in medication use research. Journal of clinical pharmacy and therapeutics. 2002;27(4):299–309. . 12174032 10.1046/j.1365-2710.2002.00430.x

[13] Angalakuditi M, Gomes J. Retrospective drug utilization review: impact of pharmacist interventions on physician prescribing. ClinicoEconomics and outcomes research: CEOR. 2011;3:105–8. doi: 10.2147/CEOR.S21789 . 21935338 10.2147/CEOR.S21789 PMC3169980

[14] Kim DS, Huh S, Lee S. Potentially inappropriate medication use at ambulatory care visits by elderly patients covered by National Health Insurance in Korea. International journal of clinical pharmacology and therapeutics. 2015;53(10):819–27. doi: 10.5414/CP202429 . 26308177 10.5414/CP202429

[15] Song I, Shin HN, Shin JY. Decrease in use of contraindicated drugs with automated alerts in children. Pediatrics international: official journal of the Japan Pediatric Society. 2017 doi: 10.1111/ped.13258 . 28177180 10.1111/ped.13258

[16] Heo JH, Suh DC, Kim S, Lee EK. Evaluation of the pilot program on the real-time drug utilization review system in South Korea. International journal of medical informatics. 2013;82(10):987–95. doi: 10.1016/j.ijmedinf.2013.07.001 . 23910897 10.1016/j.ijmedinf.2013.07.001

[17] Yu YM, Shin WG, Lee JY, Choi SA, Jo YH, Youn SJ, et al Patterns of Adverse Drug Reactions in Different Age Groups: Analysis of Spontaneous Reports by Community Pharmacists. PloS one. 2015;10(7):e0132916 doi: 10.1371/journal.pone.0132916 . 26172050 10.1371/journal.pone.0132916 PMC4501755

[18] American Geriatrics Society Beers Criteria Update Expert P. American Geriatrics Society updated Beers Criteria for potentially inappropriate medication use in older adults. Journal of the American Geriatrics Society. 2012;60(4):616–31. doi: 10.1111/j.1532-5415.2012.03923.x . 22376048 10.1111/j.1532-5415.2012.03923.x PMC3571677

[19] Zhan C, Sangl J, Bierman AS, Miller MR, Friedman B, Wickizer SW, et al Potentially inappropriate medication use in the community-dwelling elderly: findings from the 1996 Medical Expenditure Panel Survey. Jama. 2001;286(22):2823–9. . 11735757 10.1001/jama.286.22.2823

[20] O’Mahony D, Gallegher P, Ryan C, Byrne C, Hamilton H, Barry P, et al STOPP & START criteria: A new approach to detecting potentially inappropriate prescribing in old age. European Geriatric Medicine. 2010;1(1):45–51. http://dx.doi.org/10.1016/j.eurger.2010.01.007.

[21] Patel H, Bell D, Molokhia M, Srishanmuganathan J, Patel M, Car J, et al Trends in hospital admissions for adverse drug reactions in England: analysis of national hospital episode statistics 1998–2005. BMC clinical pharmacology. 2007;7:9 doi: 10.1186/1472-6904-7-9 . 17894876 10.1186/1472-6904-7-9 PMC2093926

[22] Hohl CM, Karpov A, Reddekopp L, Doyle-Waters M, Stausberg J. ICD-10 codes used to identify adverse drug events in administrative data: a systematic review. Journal of the American Medical Informatics Association: JAMIA. 2014;21(3):547–57. doi: 10.1136/amiajnl-2013-002116 . 24222671 10.1136/amiajnl-2013-002116 PMC3994866

