# Supplementary material for: Evaluation of preventable adverse drug reactions by implementation of the nationwide network of prospective drug utilization review program in Korea
Source: PLoS One. 2018 Apr 11;13(4):e0195434. doi: 10.1371/journal.pone.0195434 (PMC5895028; doi:10.1371/journal.pone.0195434)
Supplement: S1 Table — (DOCX) [file pone.0195434.s001.docx]

Table S1. List of medications under the criterion “precaution for use in the elderly”

| Number | Generic name/non-proprietary name | Dosage form |
| --- | --- | --- |
| 1 | Chlordiazepoxide | Tablet |
| 2 | Chlordiazepoxide/Clidinium | Tablet |
| 3 | Clobazam | Tablet |
| 4 | Clonazepam | Tablet |
| 5 | Clorazepate | Capsule |
| 6 | Diazepam | Tablet, parenteral injection |
| 7 | Ethyl loflazepate | Tablet |
| 8 | Flunitrazepam | Tablet |
| 9 | Flurazepam | Tablet |
| 10 | Mexazolam | Tablet |
| 11 | Pinazepam | Capsule |
| 12 | Quazepam | Tablet |
| 13 | Amitriptyline | Tablet |
| 14 | Amoxapine | Tablet |
| 15 | Clomipramine | Capsule |
| 16 | Dothiepin hydrochloride (Dosulepin) | Capsule |
| 17 | Imipramine | Tablet |
| 18 | Nortriptyline | Tablet |
| 19 | Quinupramine | Tablet |
